# Effects of biodegradable film mulching on yield, grain quality, and soil metabolic characteristics of water-saving and drought-resistant rice

**DOI:** 10.3389/fpls.2026.1847331

**Published:** 2026-06-02

**Authors:** Shikun Liu, Guangjie Zheng, Xianxian Zhang, Danping Hou, Qingyu Bi, Jinsong Tan, Zaochang Liu, Guolan Liu, Zhihai Wu, Junguo Bi, Lijun Luo

**Affiliations:** 1 College of Agronomy, Jilin Agricultural University, Changchun, China; 2 Shanghai Agrobiological Gene Center, Shanghai, China; 3 Eco-Environmental Protection Research Institute, Shanghai Academy of Agricultural Sciences, Shanghai, China; 4 Tongliao Academy of Agricultural and Animal Husbandry Sciences, Tongliao, China

**Keywords:** water-saving and drought-resistant rice, biodegradable film mulching cultivation, yield, quality, soil metabolome

## Abstract

**Introduction:**

Film mulching is an effective water-saving cultivation technique, but its regulatory mechanism on yield and grain quality of water-saving and drought-resistant rice remains unclear. This study aims to investigate how biodegradable film mulching affects rice performance and rhizosphere soil metabolism under non-flooded conditions.

**Methods:**

Field experiments were conducted in 2021 and 2022 using the water-saving and drought-resistant rice cultivar Hanyou 73 (HY73). Three cultivation treatments were applied: traditional flooded cultivation (TF), non-flooded unmulched cultivation (UM), and non-flooded biodegradable film mulching cultivation (BM). Yield components, grain quality parameters, and key enzyme activities involved in starch metabolism (SuSase, AGPase, StSase) were measured. A pot experiment was performed in 2023 to analyze rhizosphere soil metabolites using untargeted metabolomics.

**Results:**

Compared with UM, BM significantly increased rice yield by 47.5% (2021) and 49.0% (2022), reaching levels comparable to TF with no significant difference. The UM treatment severely degraded grain quality: head milled rice rate decreased by 4.04% (2021), eating quality score dropped by 12.62% (2022), amylose content declined by 16.07% (2022), and chalky grain rate increased by 57.71% (2022) relative to TF. During the early grain-filling stage, SuSase, AGPase, and StSase activities in UM grains were significantly lower than those in TF and BM, though they showed compensatory increases at later stages. Metabolomics analysis revealed that BM regulated root microecology by reshaping the composition of soil fatty acids and other metabolites.

**Discussion:**

Biodegradable film mulching enhances yield and preserves grain quality of water-saving and drought-resistant rice, primarily by optimizing starch synthesis enzyme activities during early grain filling and modulating rhizosphere soil metabolic profiles. These findings provide a theoretical basis for promoting sustainable and efficient rice cultivation using film mulching practices.

## 1 Introduction

Rice (*Oryza sativa* L.) is a pivotal food crop globally, providing the main food source for more than half of the world’s population (Ashari et al., 2024; Kamble et al., 2022; Xing et al., 2022). As the world’s largest rice producer, China has long maintained a leading position in global rice production (Tang et al., 2022). However, traditional rice cultivation in China and globally relies heavily on the flooded farming model, which accounts for approximately 90% of global rice production. This cultivation system is highly water-intensive (Belder et al., 2005) and contributes substantially to greenhouse gas emissions due to the anaerobic environment it creates. Notably, methane emissions from rice paddy account for about 48% of the total anthropogenic emissions (Li et al., 2014; Qian et al., 2022). Given that China’s per capita water availability is only 1/4 of the world’s average, the conflict between the traditional cultivation model, growing water scarcity, and the carbon neutrality goal is becoming increasingly severe. This underscores the urgent need for innovative cultivation practices to support the sustainable development of rice production.

Water-saving and drought-resistant rice is a novel variety that integrates the desired characteristics of upland rice, such as water-saving, drought resistance, and suitability for direct seeding (Bi et al., 2021). Compared to conventional rice varieties, it offers superior drought resistance and water-saving benefits; compared to upland rice, it delivers higher yield and better grain quality. When cultivated in paddy fields, it does not require flooding and can be managed under moist conditions throughout the growing period, saving more than 50% of water compared to traditional flooded systems. In dryland, it can be grown using dry direct seeding and managed with minimal irrigation, requiring water only during critical growth stages and eliminating the need to maintain a standing water layer. The large-scale adoption of water-saving and drought-resistant rice holds great potential for reducing the dependence of rice on freshwater resources and advancing environmentally sustainable rice production (Zhang et al., 2022).

Plastic film has become an important agricultural input in modern crop production. Since its first application in Japan in 1951, the use of plastic film has gradually expanded to countries such as the United States, Italy, and France. China introduced plastic film mulching technology in 1978 and has since developed it extensively over the past 50 years. By 2022, the plastic film coverage in China had reached 1.75 × 10^7^ hm², with a total usage of 1342 kt, accounting for 80% of the global usage and ranking first worldwide (Yang H. G. et al., 2023). The rice film mulching dry cultivation technology is an innovative cultivation technique that can effectively reduce irrigation frequency by limiting soil surface water evaporation. At the same time, this technology can increase soil temperature, promote nutrient uptake by rice roots, and effectively suppress weed growth, thereby reducing the competition between weeds and rice and creating favorable conditions for rice growth (Zhang et al., 2024; Zhao et al., 2024). However, after the maturity of rice and mechanized harvesting, the recycling of traditional plastic film has become a new challenge, making plastic film incompatible with rice film cultivation technology. This has led to the emergence of biodegradable film, poly (adipic acid/butylene terephthalate) (PBAT) is currently one of the better biodegradable materials in the market, which has been applied in various crops. Its biodegradable properties can effectively solve the problem of film recycling, but whether it affects the safety of soil metabolism is still unknown (Yang C. et al., 2023; Sun et al., 2025; Wang et al., 2025);.

The soil metabolome is a key mediator of soil-plant interactions. The dynamic changes in small-molecule metabolites such as fatty acids, carbohydrates, amino acids, etc. can directly reflect the synergistic relationship between soil microecological functions and crop growth (Delory et al., 2024). Film mulching dry cultivation has the potential to reshape the soil metabolic network by changing soil temperature, water availability, and microbial community structure. These changes can, in turn, affect root nutrient uptake, hormone signal transduction, and grain development (Thangamuthu and Dananjeyan, 2020; Zhang et al., 2019). In addition, film mulching may promote the accumulation of soil unsaturated fatty acids, enhance drought resistance by regulating root cell membrane fluidity, or affect soil nutrient availability by modulating carbon and nitrogen metabolic pathways.

At present, research related to rice mulching mostly focuses on the effects of agricultural film on soil physicochemical properties and soil pollution. However, research on how agricultural film indirectly improves the physiological activity, grain filling, and quality formation of aboveground rice plants in arid areas has not been in-depth. Therefore, the present study employed the water-saving and drought-resistant rice variety Hanyou 73 (HY73) and applied three cultivation treatments: non-flooded unmulched cultivation (UM), non-flooded biodegradable film mulching cultivation (BM), and traditional flooding cultivation (TF). Through field experiments and soil metabolomics analysis, the study systematically investigated: (1) the regulatory effect of film mulching on yield components and grain quality; (2) the dynamic changes in key enzyme activities involved in grain starch metabolism; and (3) the influence of film mulching on the composition and metabolic pathways of rhizosphere soil metabolites. The findings provide a theoretical foundation for optimizing the efficient and sustainable cultivation of water-saving and drought-resistant rice.

## 2 Materials and methods

### 2.1 Test materials and experimental site

Field experiments were carried out in 2021 and 2022 at the Zhuanghang Comprehensive Test Station of Shanghai Academy of Agricultural Sciences (30°53′N, 121°23′E). This site is located in a subtropical monsoon climate zone, with an average annual precipitation of 1114 mm, a frost-free period of 235 days, and sandy loam soil with a pH of 6.8 and organic matter content of 1.82%. A supplementary pot experiment was conducted in a portable greenhouse in 2023, and the rice variety used in all experiments was the water-saving and drought-resistant rice named Hanyou 73 (HY 73).

### 2.2 Experimental design

Field experiments were conducted using a randomized block design with three cultivation treatments. TF treatment (traditional flooded cultivation): a water layer was maintained from the beginning of the water management period, alternating dry and wet was applied after the tillering stage, and water was withheld one week before harvest. BM treatment (non-flooded biodegradable film mulching cultivation): plots were covered with 0.01 mm thick fully biodegradable film, with a degradation cycle of 50–120 days. After laying the film, holes were punched at a spacing of 20 cm × 23 cm, and 3 seeds were sown per hole. UM treatment (non-flooded and unmulched cultivation): bottom water was applied before sowing. Each treatment had 3 replicates, with a plot size of 6 m × 10 m. Dry direct seeding was conducted on May 15 in 2021 and June 27 in 2022. Fertilization was applied once as a basal application at the following rates: 150 kg/ha of pure nitrogen (N), 75 kg/ha of P_2_O^5^, and 120 kg/ha of K_2_O.

A pot experiment was simultaneously conducted in a mobile greenhouse in 2023. Each treatment consisted of 100 pots, and each pot was filled with 10 kg of sieved dry soil. Three rice plants were grown per pot via dry direct seeding. Fertilization was applied as follows: a basal compound fertilizer (N:P:K = 16:16:16) at 1 g per kg soil, a tillering fertilizer of 0.05 g N per kg soil and 0.02 g K_2_O per kg soil, and a panicle fertilizer of 0.05 g N per kg soil. Water management simulated the field experiment with controlled adjustments for uniform conditions: the TF treatment maintained a 3–5 cm standing water layer throughout the growing period. For the BM and UM treatments, soil relative water content was maintained at 70 - 90% by regular weighing and replenishing water, with rain exclusion to eliminate environmental fluctuations. Field trials focused on yield and grain quality assessments, while the pot experiment provided controlled rhizosphere soil samples for metabolomics analysis. The combined field–pot approach linked agronomic performance to soil metabolic mechanisms under mulching.

### 2.3 Yield and yield components

At maturity, 20 planting holes were randomly selected from each plot to assess the number of effective panicles. Among these, 5 holes were further selected for laboratory analysis to determine the number of grains per panicle, seed setting rate, and 1000-grain weight. For yield measurement, a 5m² area, excluding the border rows to minimize edge effect, was harvested, and the grain yield was adjusted to a standard 14% moisture content.

### 2.4 Grain quality

After harvesting, rice grains were stored at room temperature for 3 months prior to quality assessment. Processing quality, appearance quality, cooking quality, and eating quality were evaluated according to national rice quality standards (GB/T 17891-2021).

### 2.5 Key enzyme activities in grains

Three hundred stems were marked at the heading stage. Panicles (20-30) were collected on days 5, 10, 15, 20, 25, 30, 35, and 40 after heading, rapidly frozen in liquid nitrogen, and stored at -80°C for analysis. The activities of key enzymes involved in starch metabolism including starch branching enzyme (SBE), starch synthase (StSase), sucrose synthase (SuSase), and adenosine diphosphate glucose pyrophosphorylase (AGPase) were measured by using assay kits according to the manufacturer’s instructions (Beijing Jin Zhi Yan Biotechnology Co., Ltd., China).

### 2.6 Soil metabolite analysis

Soil samples were collected using a specialized soil drill from the 0–15 cm soil layer at the crop harvest stage. For each treatment, soil samples from 5 pots were combined into one composite sample, which was then divided into 3 biological replicates, resulting in a total of 9 test samples. Metabolomics analysis was performed by Shanghai Omicsspace Biotech Co., Ltd. (Minhang District, Shanghai, China). Untargeted metabolomics profiling was conducted using a liquid chromatography-tandem mass spectrometry (LC-MS/MS) platform. Peak alignment and metabolite identification were conducted via MS-DIAL software. Based on the R language platform, multivariate statistical methods such as Principal Component Analysis (PCA) and Orthogonal Partial Least Squares Discriminant Analysis (OPLS-DA) were employed for data analysis and identification of metabolites. Annotation was conducted using the Human Metabolome Database (HMDB) and Kyoto Encyclopedia of Genes and Genomes (KEGG) databases, with a mass error threshold of ±10 ppm. Different metabolites were defined by a fold change (FC) ≥1.5 or ≤0.67 and P<0.05, following standard protocols for metabolomics data analysis (Goodacre et al., 2004).

### 2.7 Statistical analysis

Data were analyzed using SPSS 26.0 for agronomic traits and MetaboAnalyst 5.0 for metabolomics data. For yield and grain quality data, a two - way analysis of variance (ANOVA) was performed with cultivation method and year as fixed factors, followed by the least significant difference (LSD) test for post - hoc multiple comparisons (P ≤ 0.05). Enzyme activity data were subjected to one - way ANOVA at each sampling time point. Metabolomics data were log - transformed and Pareto - scaled prior to multivariate analysis. Principal component analysis (PCA) and orthogonal partial least squares - discriminant analysis (OPLS - DA) were applied for pairwise comparisons (UM vs. TF, BM vs. TF, BM vs. UM). Model validity was evaluated using R^2^Y (explained variance) and Q^2^ (predictive ability) via 200 - fold permutation testing. Differential metabolites were defined by fold change (FC) ≥ 1.5 or ≤ 0.67 and P < 0.05 using the Student’s t - test.

## 3 Results

### 3.1 Effects of cultivation method on yield and yield components of Hanyou 73

Results from two-year experiments showed that, HY73 exhibited significant differences in the number of panicles, grains per panicle, seed setting rate, 1000-grain weight, and yield, under the three different treatments (**Table 1**). As shown in **Table 1**, the number of effective panicles in the UM treatment was 8.64% and 5.98% lower than that in the TF treatment, the number of grains per panicle decreased by 21.87% and 11.14%, and the seed setting rate also declined in both 2021 and 2022. In contrast, the number of effective panicles in the BM treatment increased significantly, while the 1000-grain weight remained stable. Notably, BM achieved yields comparable to TF, whereas UM exhibited yield reductions of 36.56% and 37.80% compared to TF in 2021 and 2022, respectively.

**Table 1:** Impacts of cultivation methods on the yield and yield components of Hanyou 73.

Year	Cultivation method	Panicle (10^4^/hm^2^)	Spikelet per panicle	Grain filling (%)	1000-grain weight (g)	Grain yield (t/hm^2^)
2021	TF	252.2 ± 10.8b	158.2 ± 10.3a	84.5 ± 2.1a	29.5 ± 0.6a	9.3 ± 0.6a
	UM	230.4 ± 13.7c	123.6 ± 14.5c	80.1 ± 1.7b	28.3 ± 0.2b	5.9 ± 1.1c
	BM	276.1 ± 12.6a	139.1 ± 8.6b	81.6 ± 1.9b	29.8 ± 0.8a	8.7 ± 0.4a
2022	TF	254.3 ± 7.6b	160.2 ± 6.8a	73.9 ± 1.3a	29.0 ± 0.6a	8.2 ± 0.9a
	UM	239.1 ± 6.8c	127.9 ± 5.2c	65.8 ± 1.0c	27.6 ± 0.4b	5.1 ± 0.8c
	BM	289.8 ± 9.7a	145.7 ± 6.5b	67.1 ± 1.5c	29.0 ± 0.3a	7.6 ± 0.4a

TF, Traditional flooding cultivation; UM, Non-flooded unmulched cultivation; BM, Non-flooded biodegradable film mulching cultivation. Different lowercase letters in the same column indicate the significant difference under different cultivation methods in the same year at the 0.05 level.

### 3.2 Effects of cultivation method on grain quality of Hanyou 73

#### 3.2.1 Effects of cultivation method on processing quality of Hanyou 73

The head milled rice rate under the UM treatment was 4.04% and 3.61% lower than that of the TF treatment in 2021 and 2022, respectively, and 5.07% and 2.95% lower than that of the BM in the same years (**Table 2**). No significant differences were observed among the treatments for the other processing quality indicators.

**Table 2:** Effects of cultivation methods on processing quality of Hanyou 73.

Year	Cultivation method	Brown rice rate (%)	Milled rice rate (%)	Head milled rice rate (%)
2021	TF	79.0 ± 0.8a	73.4 ± 1.6a	64.4 ± 1.2a
	UM	78.7 ± 0.8a	72.6 ± 1.4a	61.8 ± 0.9b
	BM	79.3 ± 0.6a	73.8 ± 1.8a	65.1 ± 1.4a
2022	TF	77.6 ± 0.9a	69.4 ± 2.5a	58.1 ± 0.8a
	UM	76.5 ± 0.8a	68.3 ± 1.6a	56.0 ± 0.8b
	BM	77.0 ± 0.5a	69.0 ± 2.0a	57.7 ± 0.6a

TF, Traditional flooding cultivation; UM, Non-flooded unmulched cultivation; BM, Non-flooded biodegradable film mulching cultivation. Different lowercase letters in the same column indicate the significant difference under different cultivation methods in the same year at the 0.05 level.

#### 3.2.2 Effects of cultivation method on appearance quality of Hanyou 73

Chalky grain rate under the UM treatment increased by 68.94% in 2021 and 57.71% in 2022 compared to the TF treatment, and the BM treatment showed increases of 18.01% and 16.49%, respectively (**Table 3**). Similarly, the chalkiness under the UM treatment increased by 32.2% and 41.37% in 2021 and 2022 compared to the TF treatment, and the BM treatment showed increases of 6.78% and 3.45%, respectively.

**Table 3:** Effects of cultivation methods on appearance quality of Hanyou 73.

Year	Cultivation method	Grain length/grain width	Transparency	Chalky grain rate (%)	Chalkiness (%)
2021	TF	3.2 ± 0.2a	2.0 ± 0.3a	32.2 ± 1.7c	5.9 ± 0.8b
	UM	3.2 ± 0.1a	2.0 ± 0.2a	54.4 ± 1.0a	7.8 ± 0.5a
	BM	3.2 ± 0.2a	2.3 ± 0.3a	38.0 ± 1.3b	6.3 ± 0.4b
2022	TF	3.1 ± 0.2a	2.0 ± 0.4a	48.6 ± 0.9b	5.8 ± 0.8b
	UM	3.0 ± 0.2a	1.7 ± 0.3a	68.2 ± 3.7a	8.2 ± 1.1a
	BM	3.1 ± 0.1 a	2.0 ± 0.4a	48.0 ± 1.4b	6.0 ± 0.7b

TF, Traditional flooding cultivation; UM, Non-flooded unmulched cultivation; BM, Non-flooded biodegradable film mulching cultivation. Different lowercase letters in the same column indicate the significant difference under different cultivation methods in the same year at the 0.05 level.

#### 3.2.3 Effects of cultivation method on cooking quality of Hanyou 73

As shown in **Table 4**, the amylose content under the UM and BM treatments was 13.10% and 12.41% lower, respectively, than that in the TF treatment in 2021, and the reductions were 16.07% and 16.55% respectively, in 2022. The gel consistency under the UM and BM treatments was 5.44% and 3.74% lower than that under the TF in 2021, and 8.33%, 6.64% lower in 2022. However, the alkali spreading value of UM and BM was 48.64%, 48.64%, respectively, higher than TF in2021 and 29.55%, 58.06% higher in 2022 (**Table 4**).

**Table 4:** Effects of cultivation methods on cooking quality of Hanyou 73.

Year	Cultivation method	Amylose content (%)		Gel consistency (mm)	Alkali spreading value
2021	TF	16.8 ± 1.2a	88.3 ± 1.0a		3.7 ± 0.1b
	UM	14.6 ± 0.8b	83.5 ± 0.7c		5.5 ± 0.6a
	BM	14.1 ± 0.9b	85.0 ± 0.6b		5.5 ± 0.5a
2022	TF	14.5 ± 0.9a	82.8 ± 0.9a		3.1 ± 0.1c
	UM	12.7 ± 0.8b	75.9 ± 0.7c		4.4 ± 0.1b
	BM	12.1 ± 1.0b	77.3 ± 0.4b		4.9 ± 0.2a

TF, Traditional flooding cultivation; UM, Non-flooded unmulched cultivation; BM, Non-flooded biodegradable film mulching cultivation. Different lowercase letters in the same column indicate the significant difference under different cultivation methods in the same year at the 0.05 level.

#### 3.2.4 Effects of cultivation method on eating quality of Hanyou 73

As shown in **Table 5**, the overall rating of the UM and BM treatment was 4.76% and 2.49% lower than that of the TF in 2021 and 2022, respectively. The appearance value of the UM and BM treatments was 14.61%, 8.99% lower than that of the TF in 2021, and 20.99%, 9.88% lower in 2022. Palate value decreased by 12.20%, 4.88% for UM and BM in 2021, and by 19.74%, 17.11% in 2022. The viscosity value of the UM and BM treatment was 50.00%, 33.33% lower than TF in 2021, and 50.00%, 50.00% for both treatments in 2022. Elasticity value of the UM and BM treatment reduced by 70.97%, 38.71% in 2021 and 81.82%, 36.36% in 2022, compared to TF (**Table 5**).

**Table 5:** Effects of cultivation methods on the eating quality of Hanyou 73.

Year	Cultivation method	Overall rating	Appearance	Palate	Viscosity	Elasticity
2021	TF	88.3 ± 1.1a	8.9 ± 0.7a	8.2 ± 0.4a	0.6 ± 0.0a	3.1 ± 0.2a
	UM	84.1 ± 0.6c	7.6 ± 0.4b	7.2 ± 0.3b	0.3 ± 0.0c	0.9 ± 0.1c
	BM	86.1 ± 1.0b	8.1 ± 0.5ab	7.8 ± 0.5ab	0.4 ± 0.0b	1.9 ± 0.1b
2022	TF	83.2 ± 1.5a	8.1 ± 0.7a	7.6 ± 0.5a	0.6 ± 0.0a	3.3 ± 0.4a
	UM	72.7 ± 1.3c	6.4 ± 0.4b	6.1 ± 0.4b	0.3 ± 0.0c	0.6 ± 0.0c
	BM	77.9 ± 1.4b	7.3 ± 0.7ab	6.3 ± 0.4b	0.3 ± 0.0c	2.1 ± 0.5b

TF, Traditional flooding cultivation; UM, Non-flooded unmulched cultivation; BM, Non-flooded biodegradable film mulching cultivation. Different lowercase letters in the same column indicate the significant difference under different cultivation methods in the same year at the 0.05 level.

#### 3.2.5 Effects of cultivation methods on activities of key enzymes in sucrose-starch metabolic pathway in Grains of Hanyou 73

**Figure 1:**
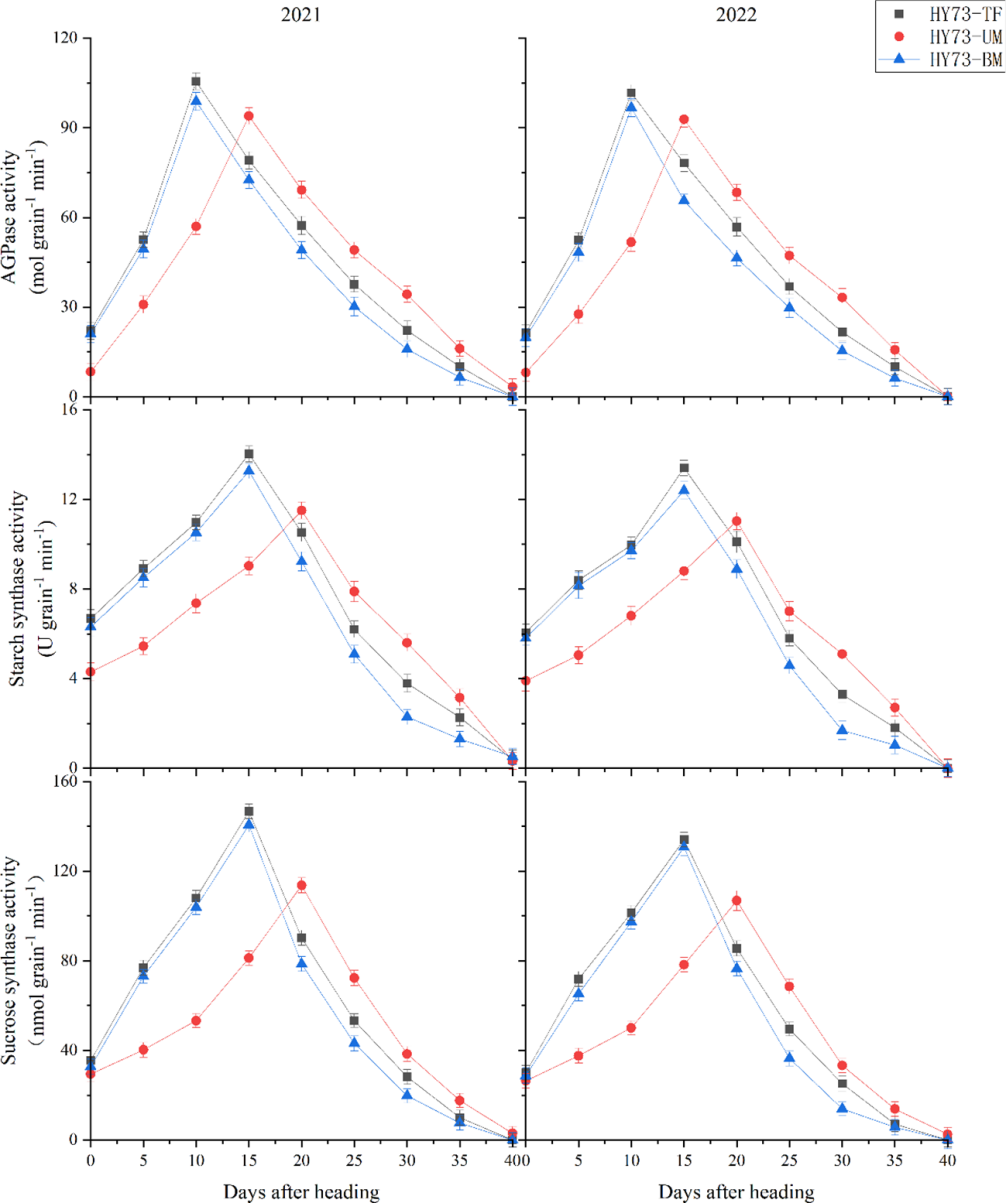
Impacts of cultivation method on the activities of AGPase, StSase, and SuSase in the Grains of Hanyou 73. TF, Traditional flooding cultivation. BM, Non-flooded biodegradable film mulching cultivation. UM, Non-flooded unmulched cultivation.

In the early grain-filling stage (5–15 days after heading), the activities of AGPase, StSase, and SuSase in the UM treatment were significantly lower than those in the TF and BM treatments, with no significant difference between BM and TF (**Figure 1**). In the middle-to-late grain-filling stage (20–25 days after heading), the activities of these enzymes in UM exceeded those in TF and BM. The peak activities of AGPase in TF and BM occurred at 10 days after heading, while StSase and SuSase peaked at 15 days after heading. In contrast, the peaks of all three enzymes in the UM treatment were delayed by 5 days, occurring at approximately 20 days after heading (**Figure 1**). Notably, the delayed but increased activities of AGPase, StSase, and SuSase in the UM treatment during the middle-to-late grain-filling stage represented an obvious physiological compensatory response to persistent soil water deficit. Under water-limited conditions, rice plants tend to up-regulate key starch-synthesizing enzymes at the late filling stage to compensate for the earlier growth inhibition, thereby maintaining grain sink strength and alleviating yield loss (Wang et al., 2023). Similar adaptive changes in assimilate transport and starch metabolism under water stress have also been widely observed in rice and other cereal crops (Chen et al., 2022; Yang et al., 2001).

### 3.3 Effects of film mulching on soil metabolomics of paddy field

#### 3.3.1 PCA analysis of metabolite composition differences

**Figure 2:**
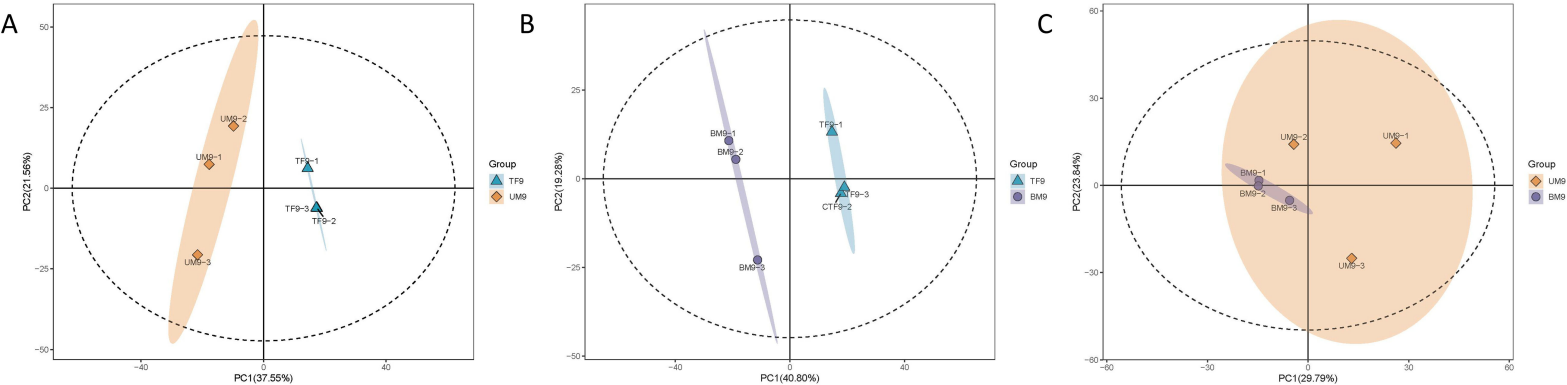
PCA analyses. **(A)** UM vs TF. **(B)** BM vs TF. **(C)** BM vs UM. TF, Traditional flooded cultivation. BM, Non-flooded biodegradable film mulching cultivation. UM, Non-flooded unmulched cultivation.

The pot experiment was designed to control environmental variability and clarify soil metabolic responses, which complemented field yield and quality data to elucidate the regulatory mechanism of biodegradable film mulching. Principal component analysis (PCA) was conducted to perform pairwise comparisons of metabolic profiles of the different treatments. Clear separation was observed between UM vs. TF and BM vs. TF, with PC1 accounting for 37.55% and 40.80% of the variation, respectively (**Figure 2**). In contrast, the separation between UM and BM was less pronounced, with a PC1 contribution rate of 29.79%, indicating smaller differences in their metabolite distributions (**Figure 2**).

#### 3.3.2 OPLS-DA analysis of metabolites

**Figure 3:**
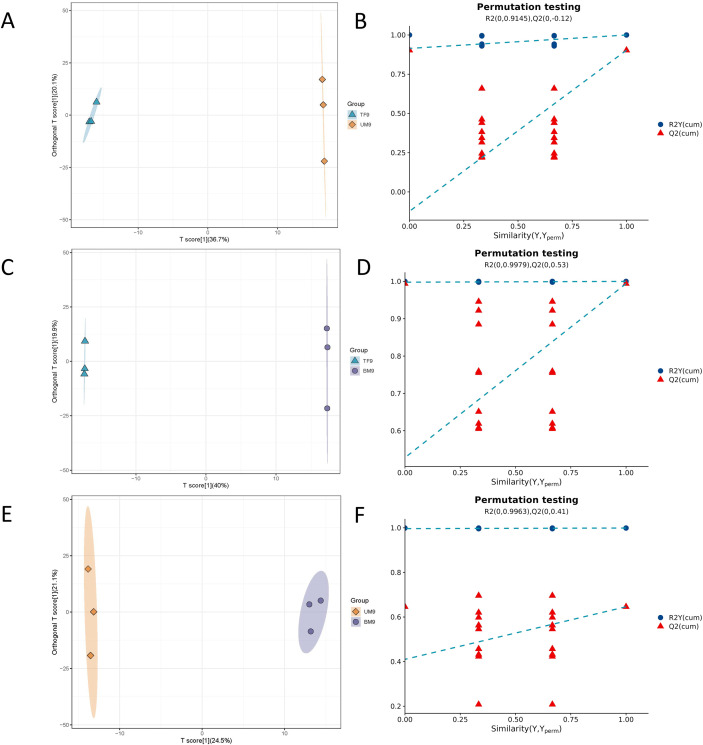
OPLS-DA analyses. **(A)** OPLS-DA score plot for comparison group UM vs TF. **(B)** OPLS-DA permutation test chart for comparison group UM vs TF **(C)** OPLS-DA score plot for comparison group BM vs TF. **(D)** OPLS-DA permutation test chart for comparison group BM vs TF. **(E)** OPLS-DA score plot for comparison group BM vs UM. **(F)** OPLS-DA permutation test chart for comparison group BM vs UM.

The UM vs. TF comparison yielded a well-fitting model with R²Y = 0.9145 and Q² = −0.12, indicating significant differences in metabolites between the two treatments. In contrast, the BM vs. UM model had a poor reliability (Q² = 0.41). Metabolites were identified based on a combination of fold change FC≥1.5 and P < 0.05 thresholds (**Figure 3**). Notably, the BM vs. UM comparison yielded a relatively low Q^2^ value of 0.41, indicating that the two treatments shared a high degree of similarity in overall soil metabolic profiles and that the model had limited predictive power for global differences. This result was consistent with the PCA showing only weak separation between BM and UM. However, the stringent threshold (FC ≥ 1.5, P < 0.05) identified 67 significantly altered metabolites, mainly involved in lipid and carbohydrate metabolism. These findings demonstrate that biodegradable film mulching did not fundamentally reshape soil metabolism but rather induced subtle, targeted changes in key metabolic pathways related to root–soil interactions. The combination of PCA separation, pathway enrichment, and consistent differential metabolite patterns across comparisons supports the reliability and validity of the study’s conclusions.

#### 3.3.3 Metabolite classification analysis

**Figure 4:**
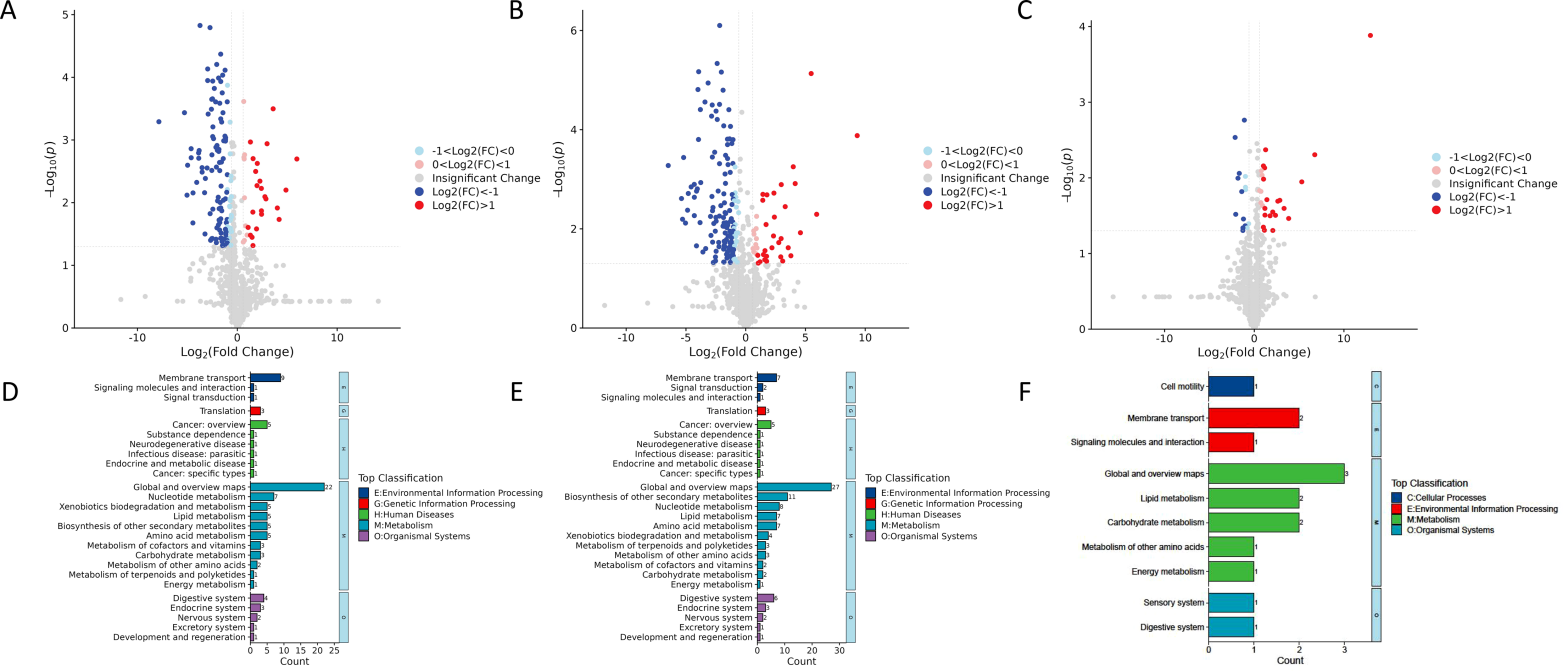
**(A–C)** Volcanoplots of upregulated and downregulated differentially expressed metabolites under different treatments: **(A)** UM vs TF, **(B)** BM vs TF, **(C)** BM vs UM. **(D–F)** Bar charts of KEGG pathway enrichment counts for second-level categories, with the comparison order as follows: **(D)** UM vs TF, **(E)** BM vs TF, **(F)** BM vs UM.

Using fold change (FC) ≥1.5 or ≤0.67 combined with a significance level of P < 0.05 as screening criteria, a total of 200 different metabolites were identified in the UM vs. TF group, including34 up-regulated, 166 down-regulated metabolites (**Figure 4A**). These metabolites were primarily enriched in the pathways related to carbohydrate metabolism and amino acid metabolism (**Figure 4D**). In the BM vs. TF group, 180 different metabolites were identified, including 40 up-regulated, and 143 down-regulated (**Figure 4B**). Eleven metabolites were enriched in the Biosynthesis of other secondary metabolites pathway was enriched with 11 (**Figure 4E**). The BM vs. UM comparison identified 67 different metabolites, of which50 were up-regulated and 17 down-regulated (**Figure 4C**), mainly associated with lipid metabolism and carbohydrate metabolism pathways (**Figure 4F**).

## 4 Discussion

In this study, the BM treatment significantly increased yield by 47.5% in 2021 and 49.0% in 2022 compared to the UM treatment, and there was no significant difference in yield between the BM and TF treatments (**Table 1**). This yield improvement may be attributed to the water retention and temperature-regulating effects of the plastic film mulching technology (Wu et al., 2017). Biodegradable film mulching can reduce soil evaporation by over 30%, maintaining soil moisture at 60%−70% of the field capacity, thereby providing an optimal water environment for root development and growth (Flory et al., 2021; Liu and Gao, 2018). Additionally, soil temperature under the film is 2−3°C higher than that of bare soil, accelerating nutrient uptake, particularly nitrogen, phosphorus, and other nutrients. These nutrients are essential for rice tillering and the formation of productive panicles, which is consistent with previous research (Arai-Sanoh et al., 2010). Furthermore, plastic film mulching suppresses weed growth, minimizing competition and enhancing the allocation of photosynthetic assimilates to grains (Ming and Chen, 2020). Notably, the 1000-grain weight under the BM treatment did not differ significantly from that under the TF treatment, indicating that film mulching did not affect grain plumpness, an important factor in maintaining yield stability under water-saving cultivation practices.

Grain quality under the UM treatment deteriorated significantly (**Tables 2**–**5**). During the early stage of grain-filling (5−15 days after heading), the activities of key enzymes involved in starch synthesis, such as AGPase and SuSase under the UM treatment, were 20%-30% lower than those under TF. This enzymatic reduction led to an insufficient supply of starch precursors (ADPG, sucrose), resulting in a loose arrangement of starch granules in endosperm cells and the development of chalkiness. The delayed peak of enzyme activity observed in UM (**Figure 1**) may be associated with drought-induced accumulation of abscisic acid (ABA), which is known to upregulate starch synthesis genes during the late grain-filling stage, a phenomenon also reported in Mediterranean ornamental plants under water stress (Toscano et al., 2019). Interestingly, enzyme activity under the UM treatment exceeded that of the TF during the later stage of grain filling (20−40 days), which could be a compensatory physiological response to drought stress (Toscano et al., 2019).

The reduction in amylose content is closely related to the inhibition of AGPase activity, an enzyme responsible for catalyzing the conversion of glucose-1-phosphate to ADPG, which is essential for starch synthesis (Li et al., 2018). Previous studies have reported increased ABA content in grains under the non-mulched dry cultivation conditions (Zhang et al., 2020). Elevated ABA content inhibits the expression of starch synthesis genes, thereby further reducing amylose synthesis efficiency (Wu et al., 2023). In contrast, the BM treatment helps maintain soil moisture, leading to an earlier and more sustainable peak in enzyme activity, which supports a balanced starch synthesis. The chalky grain rate under BM increased by only 16.5% compared to TF, which was significantly lower than that observed under UM (**Table 3**), indicating that film mulching can effectively alleviate the adverse effect of drought on grain quality (Behzadnejad et al., 2020).

Changes in soil metabolome under film mulching treatment are complex and multi-faceted, resulting from the interplay of spatial-temporal factors such as planting area, soil type, mulching time, and mulch material (Xing et al., 2022; Zhang J. R. et al., 2025). Film mulching affects soil microbial community composition, alters soil nutrients and metabolite composition, and creates complex ecological effects (Baldrian and Valášková, 2008). Some soil substances, particularly complex organic matter, require initial degradation by fungal-secreted hydrolytic enzymes and oxidoreductases before they can be further broken down and utilized by other microorganisms like bacteria (Shah et al., 2016). A decline in key fungal populations may hinder this process, disrupt nutrient cycling, thereby reducing available essential nutrients in the mulched soil (Xue et al., 2023), and potentially pose risks to soil ecosystem stability. In summary, the impact of film mulching on soil metabolomics is a multi-dimensional process shaped by a variety of biotic and abiotic factors. In future studies, the impact of rice film mulching cultivation on soil metabolome should be further explored to allow a more comprehensive understanding of its agronomic and environmental implications. Comparative studies across different regions are also needed to provide more region-specific recommendations for optimizing film mulching practices and promoting sustainable soil ecosystem management.

In the present study, PCA and OPLS-DA analyses showed that there were significant differences in soil metabolite profiles between UM vs. TF and BM vs. TF, while the metabolic differences between UM and BM were comparatively smaller. Specifically, the UM vs. TF comparison identified 200 differential metabolites, primarily enriched in the pathways of “Carbohydrate metabolism” and “Amino acid metabolism” (**Figure 4**), indicating that non-mulched dry cultivation significantly disturbed the soil carbon and nitrogen metabolic network. This is consistent with previous research findings (Manzoni et al., 2019). Reduced soil moisture changed the structure and function of the microbial community, thereby affecting the decomposition rate of carbohydrates. The BM vs. TF group identified 180 differential metabolites, with 11 enriched in the “Biosynthesis of other secondary metabolites” pathway, indicating that film mulching may improve soil microecology by regulating secondary metabolites (such as fatty acids, phenols) (Chen et al., 2024). The 2−3°C increase in soil temperature under BM likely accelerated lipid metabolism, as evidenced by the enrichment of unsaturated fatty acids (**Figure 4E**). This aligns with previous findings that soil warming promotes root membrane fluidity via fatty acid desaturation (Zhao et al., 2021). The BM vs. UM comparison identified only 67 differential metabolites, mainly associated with “lipid metabolism” and “carbohydrate metabolism”, suggesting that film mulching support efficient root–soil material exchange by subtly regulating the soil carbon metabolism process (Liu et al., 2023). In addition, the enrichment of metabolites in the “membrane transport” pathway (such as amino acid transporter-related substances) suggests that film mulching may promote the root uptake of water and nutrients by enhancing transmembrane transport capacity.

In summary, the present study demonstrates that biodegradable film mulching reshapes rhizosphere soil metabolism by modulating carbohydrate, amino acid, and lipid metabolic pathways, thereby improving root function and grain yield and quality. The fully biodegradable film applied here has a degradation cycle of 50–120 days and can be completely degraded into harmless substances under field conditions, effectively avoiding residual pollution from conventional plastic film. While our short - term results confirm that biodegradable film mulching does not disrupt soil metabolic homeostasis, long - term monitoring is still required to systematically assess the environmental fate of degradation products and their potential effects on soil microbial communities and nutrient cycling. Such information will further ensure soil metabolic safety and support the sustainable development of water - saving rice cultivation.

Regarding the biodegradable film itself, the PBAT-based material used in this study has a degradation cycle of 50–120 days, which can be completely degraded into CO_2_, H_2_O, and small organic molecules under field conditions, without the persistent residues associated with conventional plastic films. This addresses the initial concern about the safety of soil metabolism raised in the introduction. Although biodegradable mulch films are designed for complete degradation under field conditions, incomplete breakdown often generates microplastics that can alter soil microbial communities and nutrient cycling across multiple cropping cycles (Zhou et al., 2023). Long-term, multi-year field studies over multiple cropping cycles are still needed to systematically evaluate the environmental fate of degradation products, as well as their potential impacts on soil microbial community structure, nutrient transformation, and soil metabolic stability (Bandopadhyay et al., 2020; Zhang Y. et al., 2025). Such long-term assessments are essential to fully evaluate the ecological sustainability of biodegradable film mulching and ensure the safe application of this technology in water-saving rice production.

## 5 Conclusions

This study demonstrates that film mulching dry cultivation is an effective and sustainable cultivation model that simultaneously achieves water saving, high yield, and good grain quality. Film mulching dry cultivation reduced irrigation water used by more than 50% compared to TF, based on cumulative water input throughout the growing season. The observed synergistic improvement of yield and quality is attributed to the regulation of soil hydrothermal conditions, grain starch metabolic enzyme activity, and the rhizosphere soil metabolic network. These findings provide a strong theoretical foundation for promoting green and resource-efficient production of water-saving and drought-resistant rice. It is highly recommended to promote the application of fully biodegradable film mulching technology in water-scarce regions as a strategy to balance agricultural productivity and ecological sustainability. Future studies are needed to validate this cultivation technique across different soil types (e.g., clay vs. sandy loam) to optimize its application in diverse agroecosystems.

## Data Availability

The metabolomics data presented in this study have been deposited in the MetaboLights repository with the accession number MTBLS14587. The dataset can be accessed at: https://www.ebi.ac.uk/metabolights/MTBLS14587.
